# Hilar and Extrahepatic Inflammatory Pseudotumour: A Case Report and Systematic Literature Review

**DOI:** 10.7759/cureus.79727

**Published:** 2025-02-26

**Authors:** Nicole Hawkins, David Sun, Panuwat Pornkul, Kaeun Bae, Matan Ben David

**Affiliations:** 1 General Surgery, Townsville University Hospital, Townsville, AUS; 2 Upper Gastrointestinal and Hepatobiliary Surgery, Townsville University Hospital, Townsville, AUS

**Keywords:** biliary diseases, cholangiocarcinoma mimic, extrahepatic biliary obstruction, inflammatory pseudotumour, pseudotumour

## Abstract

Inflammatory pseudotumor (IPT) is a benign inflammatory lesion that is exceptionally rare in the biliary tree. Its clinical and radiological presentation mimics neoplastic disease, so diagnosis often relies on histology. Surgical resection is the mainstay of treatment. A case of histologically confirmed IPT in a 52-year-old female, successfully managed with surgical resection, is presented. A systematic literature review was conducted following Preferred Reporting Items for Systematic Reviews and Meta-Analyses (PRISMA) guidelines to identify case reports and case series of IPT involving the extrahepatic bile ducts and liver hilum. Searches of PubMed and Embase (from January 1960 to December 2024) yielded 23 original articles that met inclusion criteria. Data on clinical presentation, management, and outcomes were analyzed. A total of 33 cases of hilar and extrahepatic IPT have been reported in the literature. Obstructive jaundice was the most common presentation (79% n=26/32) accompanied by biochemical elevation of liver function tests (91% n=21/23) and bilirubin (77% n=17/22). Surgical resection was the most common treatment (82% n=27/33) with excellent outcomes and only one case of recurrence. However, the follow-up period was relatively short (median of one year). Of five cases initially treated with steroids, three were successfully managed with steroids alone. The other two cases proceeded to surgical resection due to disease progression. Serum immunoglobulin-G4 was high in successfully managed cases and not reported in failed cases. Hilar and extrahepatic IPT is a rare pathology with a similar presentation to neoplastic disease, which can make diagnosis and management challenging. Surgical resection is the mainstay of management, however, in select cases, preoperative biopsy may help avoid unnecessary surgical intervention. Further studies with extended follow-up are needed to optimize diagnostic and therapeutic strategies.

## Introduction

Inflammatory pseudotumor (IPT) is a rare, non-neoplastic inflammatory lesion that mimics malignant tumors in clinical and radiological presentations. First described by Umiker and Iverson in 1954, IPT has been reported in all organ systems [1,2]. While intrahepatic IPT accounts for approximately 0.4% of focal liver lesions treated by hepatectomy, its occurrence in the biliary tree is exceedingly rare [2-5].

IPT at the porta hepatis and extrahepatic bile ducts can present with obstructive jaundice, abdominal pain, weight loss, fever, and fatigue [2-3]. It is indistinguishable from neoplastic lesions, such as cholangiocarcinoma, on imaging (including ultrasonography, CT, and MRI) and so presents a difficult diagnostic dilemma. Most frequently, IPT is diagnosed after obtaining tissue sampling, which in hilar and extrahepatic IPT is often after patients undergo extensive resection [6].

Histopathological features of IPT include a mix of acute and chronic inflammatory cells (primarily lymphocytes and eosinophils) and collagen tissue composed of fibroblasts and myofibroblasts [7-8]. Historically, IPT and inflammatory myofibroblastic tumor (IMT) were considered a single pathological entity due to shared morphological characteristics, particularly fibroblastic-myofibroblastic proliferation. However, IMT is now recognized as a distinct entity with neoplastic potential, typically affecting younger individuals compared to IPT, which is more common in older adults. Diagnosis requires a combination of histology, immunohistochemistry, and fluorescent in situ hybridization, with a key point of difference being that immunoglobulin-G4 (IgG4) plays an important role in the pathogenesis of IPT but not IMT [9-11].

The management of IPT generally involves surgical resection, often due to diagnostic uncertainty or concerns the lesion is malignant. However, cases of spontaneous regression and resolution with systemic steroid treatment have been reported in intrahepatic IPT [12].

A case of IPT involving the porta hepatis and extrahepatic bile ducts is presented to contribute to the limited literature on this rare presentation. A systematic review of the literature was conducted to better define the clinical presentation, investigation, and management of hilar and extrahepatic bile duct IPT.

## Case presentation

A 52-year-old Caucasian woman presented to her general practitioner with perimenopausal symptoms, including insomnia and night sweats. She denied abdominal pain or weight loss. She was a nonsmoker with no significant medical history aside from an appendicectomy. Physical examination was unremarkable. Routine blood tests revealed mild liver enzyme derangement (Table 1). Hepatitis screening was negative, and serology for Cytomegalovirus and Epstein-Barr virus indicated prior exposure without acute infection.

**Table 1:** Preoperative blood tests ALP - alkaline phosphatase, AST - aspartate aminotransferase, ALT - alanine aminotransferase, GGT - gamma-glutamyl transferase, CEA - cancer embryonic antigen, CA 19.9 - cancer antigen 19.9

Laboratory Study	Value (Reference Range, Units)
Bilirubin	11 (<16 umol/L)
ALP	200 (30-115 U/L)
AST	33 (10-35 U/L)
ALT	67 (5-30 U/L)
GGT	279 (5-35 U/L)
CEA	5.2 (<5.0 ug/L)
CA 19.9	29 (<34 U/L)

**Figure 1:**
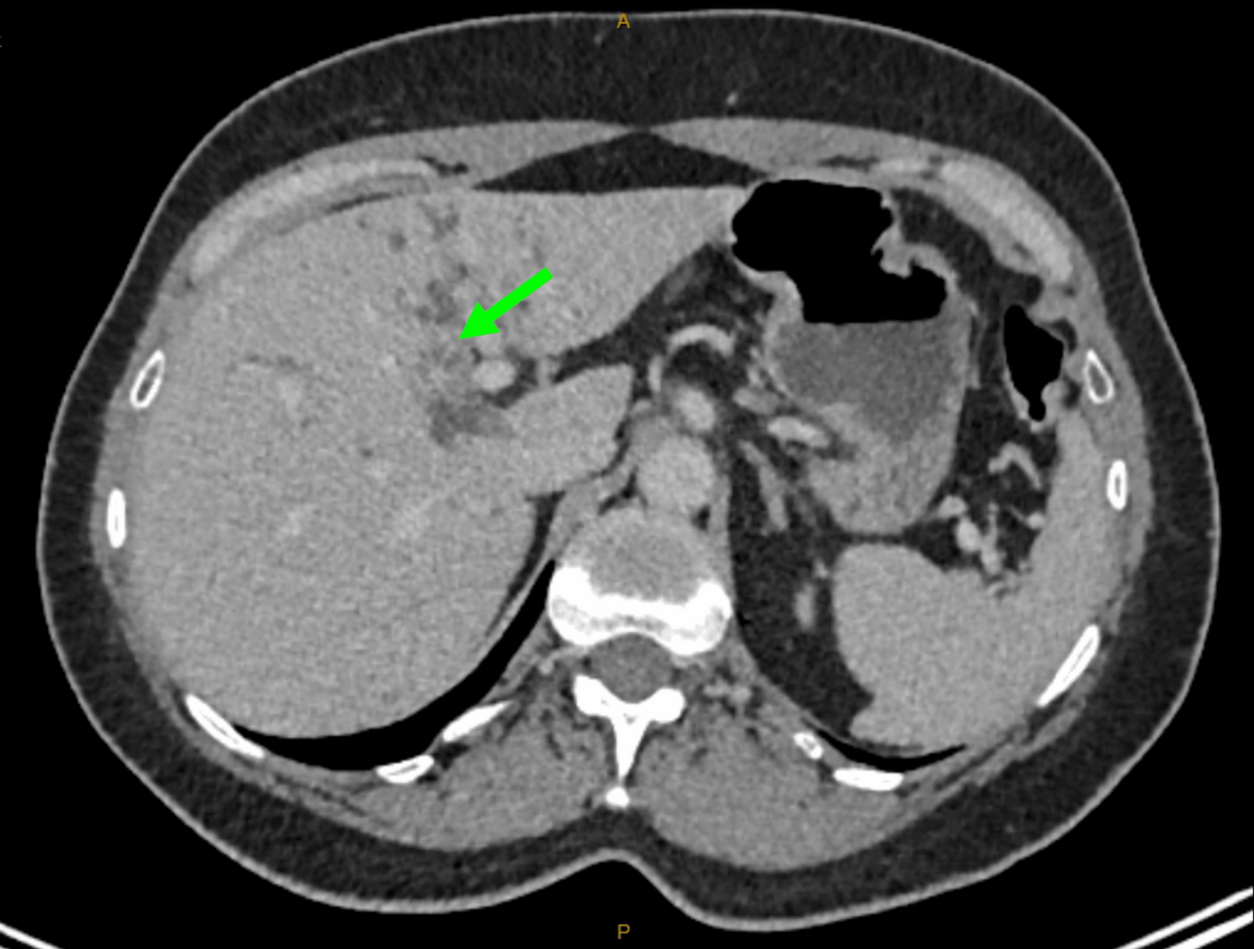
CT findings are highly suspicious for cholangiocarcinoma centered on the left lobe of the liver Green arrow: Tumor at the porta hepatis

**Figure 2:**
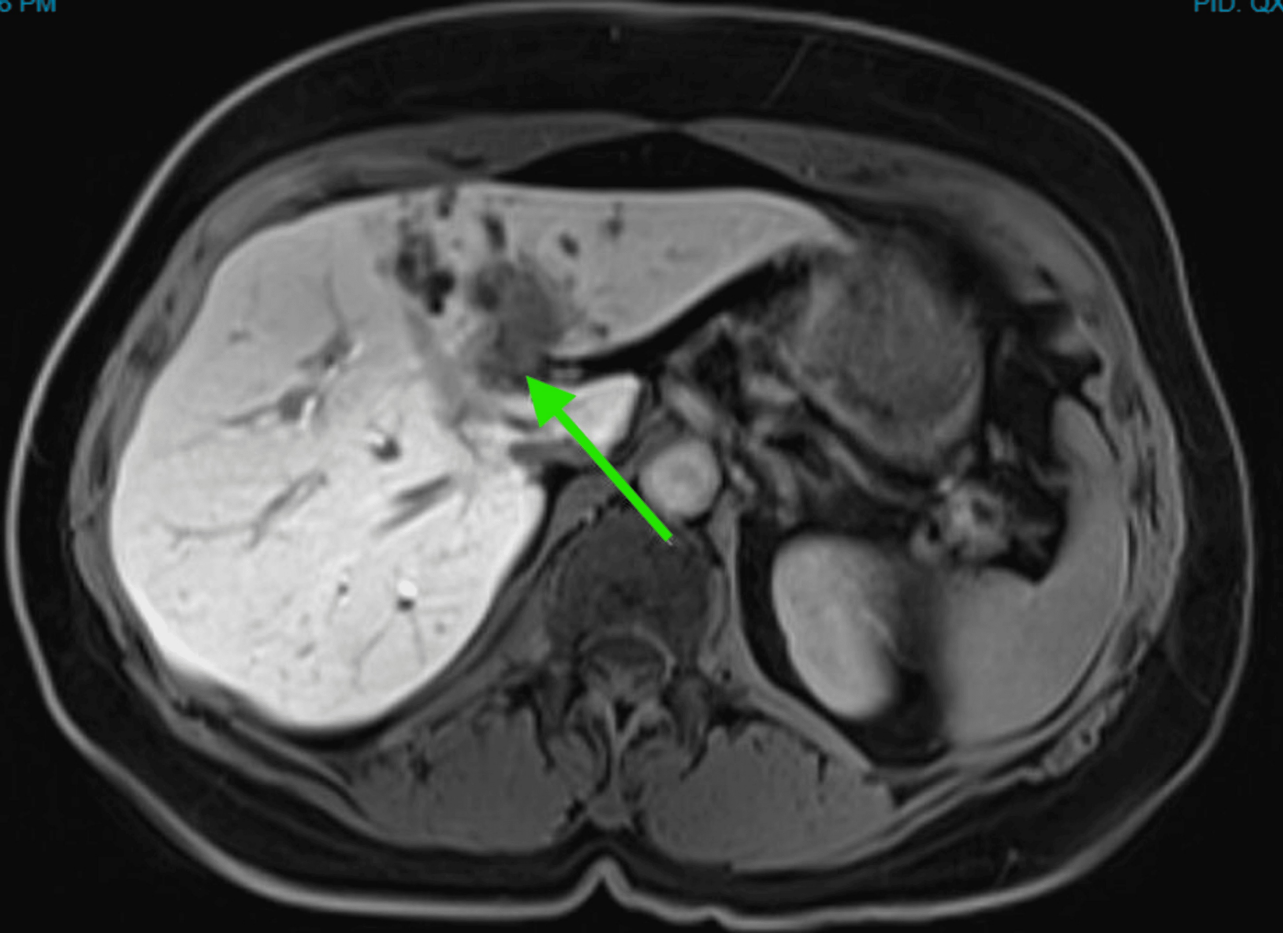
MRI demonstrated marked left lobe intrahepatic biliary dilatation with suspicious soft tissue density at the porta hepatis surrounding the left hepatic ducts Concerning for further disease was extension along the common hepatic duct and along the right hepatic duct. Green arrow: soft tissue density at the porta hepatis surrounding the left hepatic ducts

**Figure 3:**
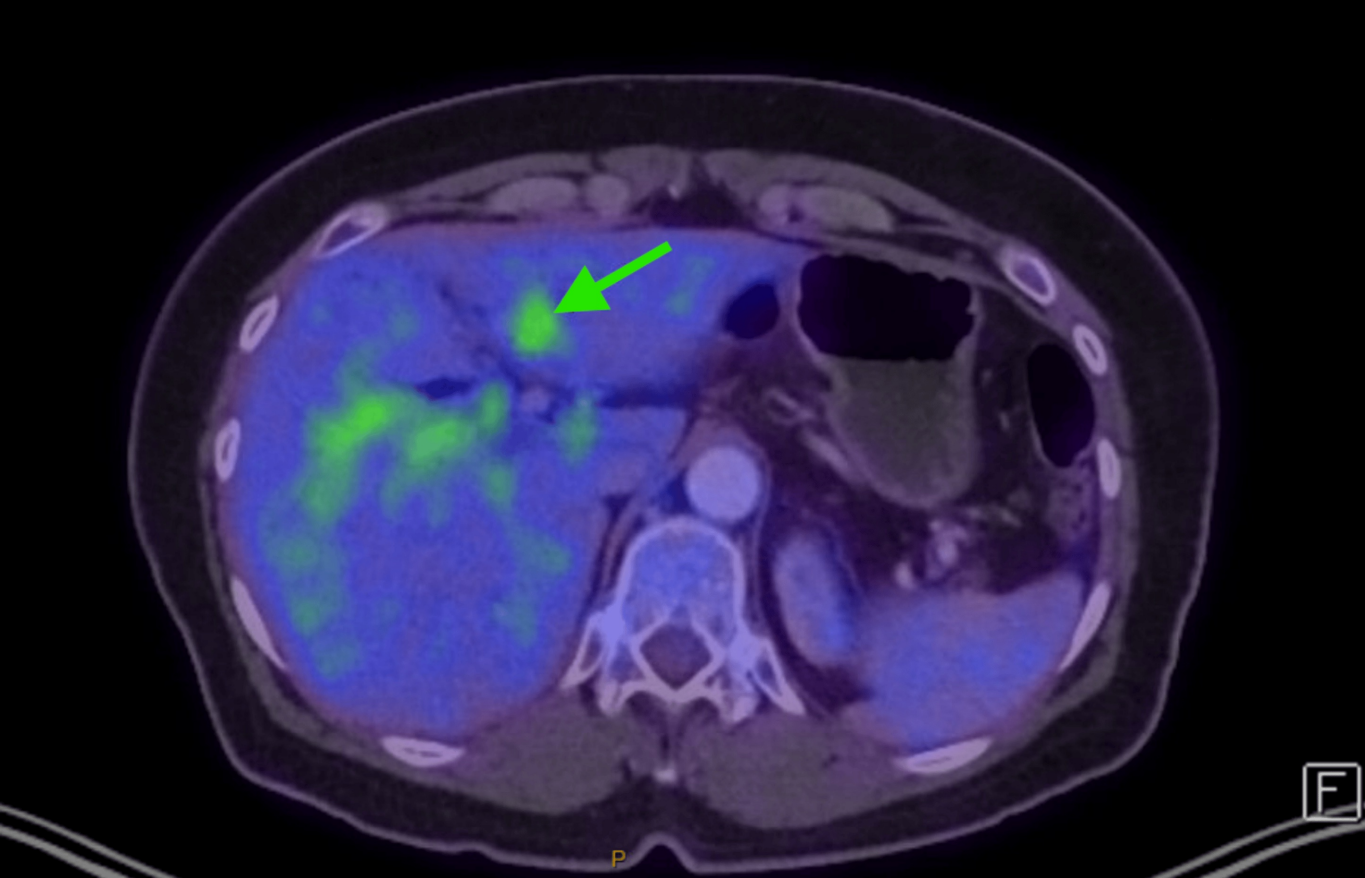
PET demonstrated a focal area of increased FDG uptake in the left lobe of the liver, which correlated closely with a hypoenhancing lesion on MRI and, therefore, was very suspicious for malignancy Green arrow: FDG uptake in the left lobe of the liver PET: positron emission tomography; FDG: fluorodeoxyglucose

A CT scan of the abdomen and pelvis showed intrahepatic biliary duct dilatation, predominantly in the left hepatic lobe, with mural enhancement involving the central hepatic ducts, common hepatic duct, and upper bile duct, raising concerns for malignancy (Figure 1). Subsequent MRI and PET imaging supported a diagnosis of hilar cholangiocarcinoma (Figures 2, 3). Tumor markers, including carcinoembryonic antigen (CEA) and cancer antigen 19-9 (CA.19.9), were unremarkable.

The case was discussed at a multidisciplinary team meeting, where the consensus was to proceed with extended resection based on imaging findings, given the difficulty of obtaining preoperative histology. The patient underwent an extended left hepatectomy with extrahepatic biliary resection and portohepatic lymphadenectomy. Intraoperative frozen section analysis of the distal bile duct margin was negative for malignancy. Her operative and postoperative courses were uneventful, and she was discharged six days after surgery.

**Figure 4:**
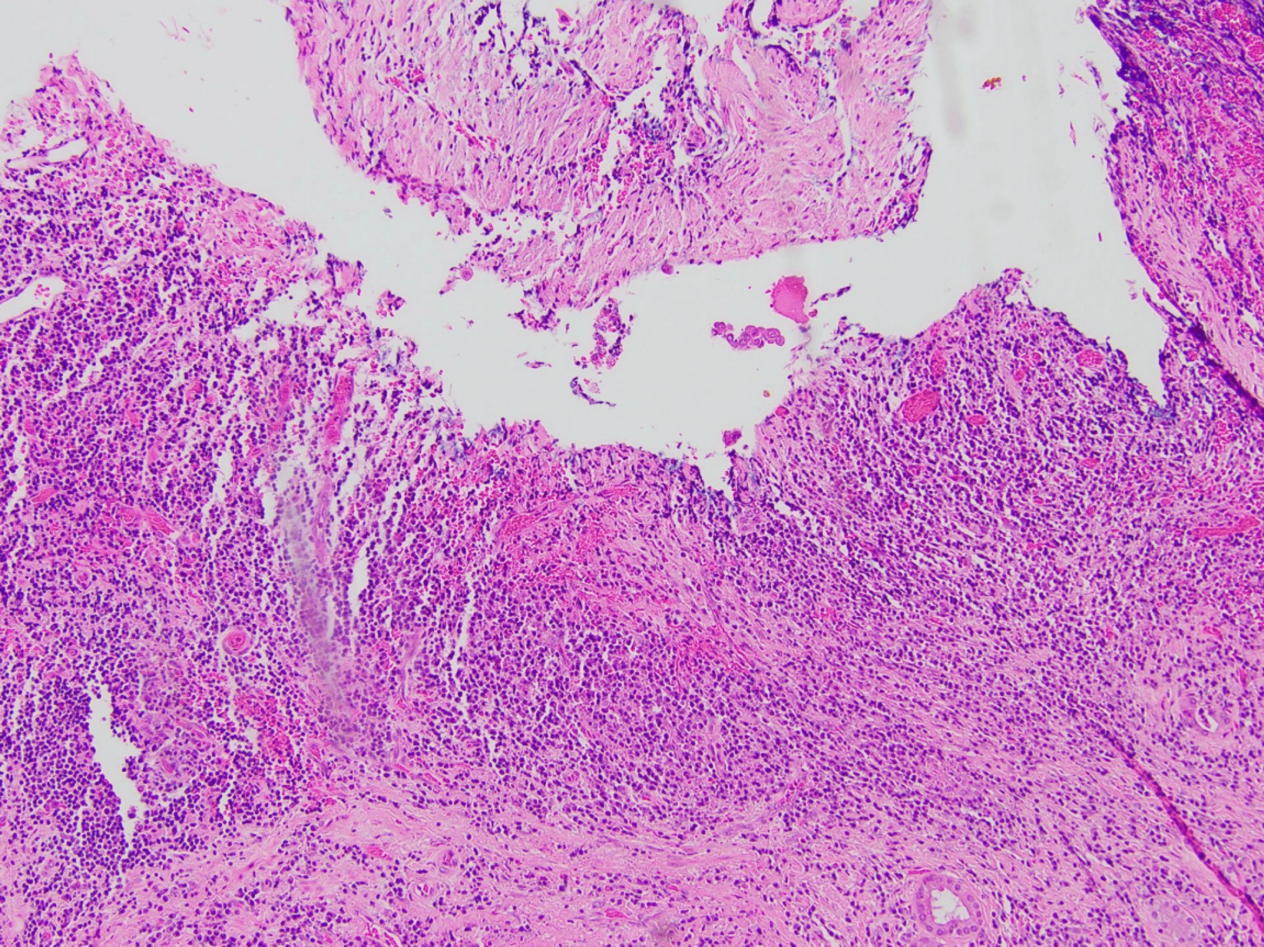
Medium power microscopy inflammatory pseudotumor at the porta hepatis

**Figure 5:**
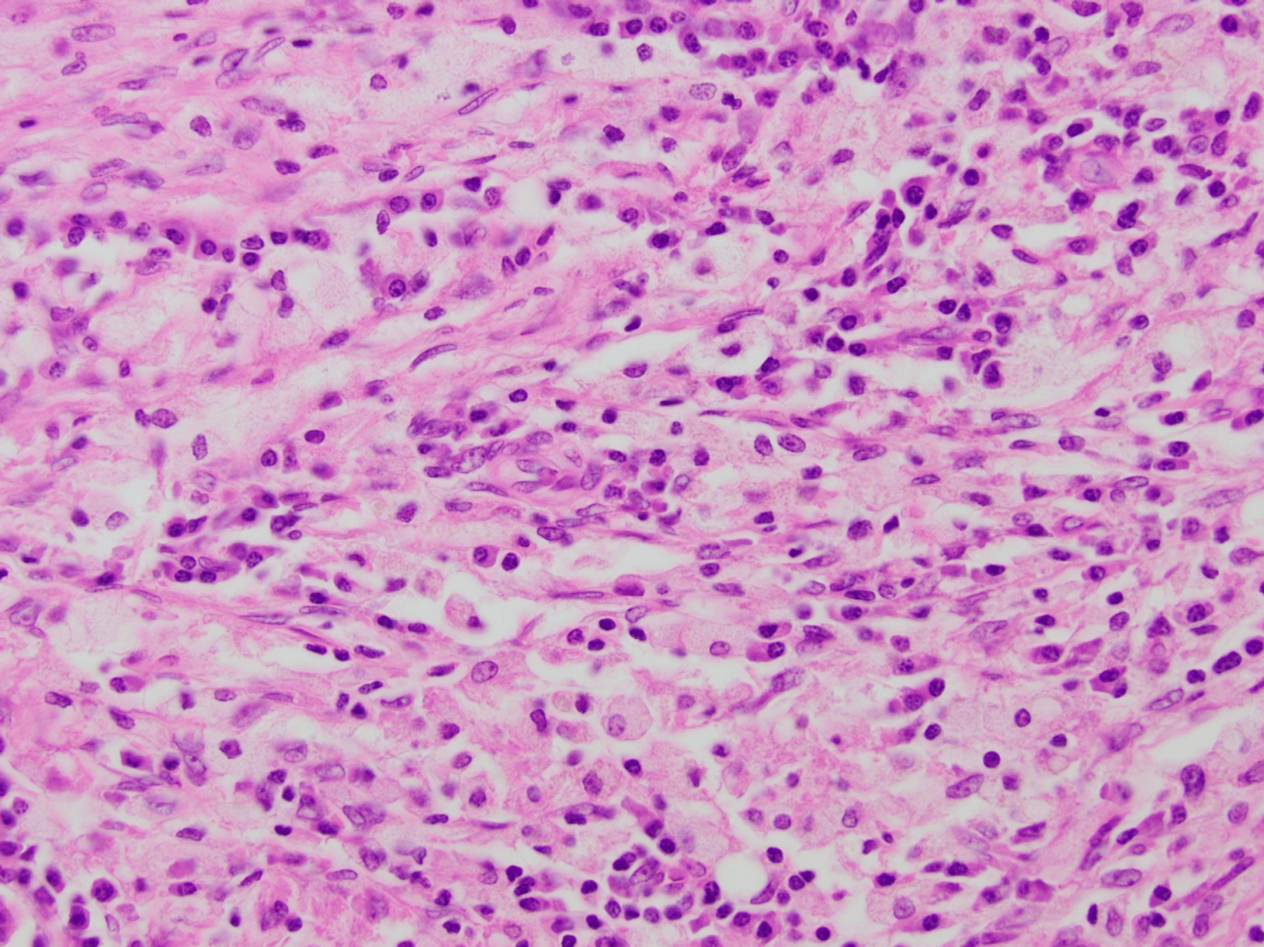
Xanthogranulomatous inflammation within the inflammatory pseudotumor at the porta hepatis

Histological examination of the resected specimen revealed an IPT of the hilum and extrahepatic bile ducts. Microscopy showed focal neutrophilic debris within the large duct lumens, edema, and dense mixed periductal inflammation, including lymphocytes, plasma cells, and granulocytes (Figure 4). Dominant histopathologic changes are centered on the extrahepatic ducts (particularly the common bile duct and common hepatic duct, with lesser involvement of the cystic duct). Focal xanthogranulomatous inflammation was also noted (Figure 5). Porta hepatis lymph nodes were free of malignancy. 

At four months postoperatively, the patient remains well, with no clinical or radiological evidence of disease recurrence. 

## Discussion

Systematic literature review

Methods

A systematic literature review was conducted following Preferred Reporting Items for Systematic Reviews and Meta-Analyses (PRISMA). Case reports and case series of patients with a histologically confirmed inflammatory pseudotumor in the extrahepatic bile ducts (specifically common hepatic, common bile, and cystic ducts) or liver hilum (with involvement of the intrahepatic bile ducts) of any age, including details on clinical presentation and management, were included.

Electronic searches of PubMed and Embase databases were conducted including articles from January 1960 to December 2024. Search terms were deliberately broad due to the rarity of this pathology and included (1) pseudotumor and (2) 'biliary' OR 'bile duct' OR 'common bile duct' OR 'cystic duct' OR 'hepatic duct' OR 'CBD' OR 'hilar’. Articles were screened by title and abstract by two independent authors. Abstracts and full text were then retrieved and further screened. Additional papers were identified through reference checks of published articles. Only articles written in English were included.

Exclusion Criteria 

Studies that referred to the pathology as exclusively IMT or IMT and IPT interchangeably were excluded (except for studies that reference IMT as a synonym of IPT on a single occasion and where histology clearly reported IPT). Cases of intrahepatic, gallbladder, or pancreatic IPT without involvement of the extrahepatic bile ducts or porta hepatis were also excluded.

Results

**Figure 6:**
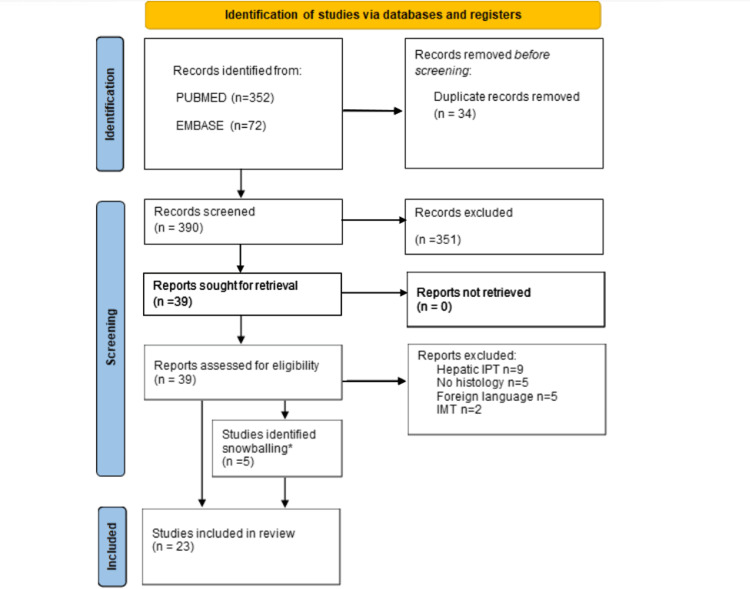
PRISMA 2020 flow diagram for new systematic reviews * Two studies were not able to be retrieved for review PRISMA: Preferred Reporting Items for Systematic Reviews and Meta-Analyses

Using the search term above, 390 articles were identified from the PubMed and Embase databases. After screening titles and abstracts, 37 articles were assessed for eligibility. After exclusions, 23 papers were included (Figure 6). Two articles identified from reference checks were not able to be retrieved for full-text review and so were excluded [13-14].

A summary of the literature review findings is displayed in Table 2. Twenty-three studies, 5 case series and 18 case reports, reported a total of 32 cases of pseudotumors at the liver hilum and/or extrahepatic bile ducts. Including our current case, the median and average age at presentation were 56 and 45 years, respectively (range 6 years to 71 years), and there were similar numbers of males and females (17 and 15 cases, respectively).

**Table 2:** Cases of hilar or extrahepatic bile duct IPT identified on systematic review Sources: [3,5,6,15-34] LFT – liver function tests, CBD – common bile duct, PI – pancreatic insufficiency, CHD – common hepatic duct, CD – cystic duct, RUQ – right upper quadrant, GB – gallbladder, IPT – inflammatory pseudotumor, US – ultrasound, ERCP – endoscopic retrograde cholangiopancreatography, EUS-FNB – endoscopic ultrasound fine-needle biopsy, N/A - not applicable * Denotes case series

Author	Age and Presentation	Serum Bilirubin/LFT	Location of IPT	Preoperative Sampling	Preoperative Diagnosis	Treatment	Follow-up
Haith 1964 [15]	6M vomiting, diarrhea, fever, obstructive jaundice, and anorexia/weight loss	High/High	CBD	-	Choledochal cyst	Surgical resection	5 months, no recurrence
Stamatakis 1979 [16]	13F abdominal pain and obstructive jaundice	High/High	Hilar + CHD + CBD	-	-	Surgical resection	21 months, no recurrence
Jaffri 1983 [17]	41M RUQ pain and diarrhoea	High/High	CBD	-	-	Surgical resection	2 years, no recurrence
Heneghan 1984 [18]	8F abdominal pain, obstructive jaundice, lethargy, anorexia	High/High	Hilar	-	Inflammatory mass vs infiltrating cancer	Surgical resection + transplantation	1 year, no recurrence
Ikeda 1990 [19]	43M obstructive jaundice, fever, weight loss	Normal/High	Hilar + CHD + CBD + CD + GB	-	-	Surgical resection	7 months, no recurrence
Pokorny 1991 [20]	43M nausea, epigastric pain, and obstructive jaundice	High/High	Right hepatic lobe + Hilar + CBD	Core biopsy liver - IPT	-	Biliary stenting	5 years, no change in serial imaging
Nakanuma 1994* [21]	59M RUQ pain	High/ -	Hilar	-	Cholangiocarcinoma	Surgical resection	-
Nakanuma 1994* [21]	67M malaise	High/ -	Hilar	-	Cholangiocarcinoma	Surgical resection	-
Nakanuma 1994* [21]	52M obstructive jaundice + shock	High/High	Hilar	-	Cholangiocarcinoma	Death prior to treatment	-
Fukushima 1997 [22]	58F deranged LFT	Normal/High	CBD	Core biopsy liver - inconclusive	Cholangiocarcinoma	Surgical resection	-
Nonomura 1997 [23]	64M anemia, elevated thymol turbidity, mass found on US	- /Normal	Hilar	-	Cholangiocarcinoma	Surgical resection	11 months, no recurrence
Shanti 2001* [24]	57F nausea, vomiting, upper abdominal pain, and obstructive jaundice	High/High	Hilar	-	Cholangiocarcinoma	Surgical resection	6 months, no recurrence
Worley 2001 [25]	57F nausea, abdominal pain, anorexia/weight loss, and obstructive jaundice	High/High	Hilar + CHD	-	Cholangiocarcinoma	Surgical resection	-
Kaneko 2001 [5]*	6M fever and obstructive jaundice	High/High	Hilar	-	-	Surgical biopsy and subsequent stenting. In two years and nine months, IPT progressed despite steroids and proceeded to surgical resection.	14 months, no recurrence
Sobesky 2003 [26]	51F obstructive jaundice and RUQ pain	High/High	CBD	ERCP biopsy - inconclusive	Pancreatic adenocarcinoma	Surgical resection	2 years, no recurrence
Ueda 2003 [27]	7M fever and obstructive jaundice	High/High	Hilar	Core biopsy - IPT	IPT	Failed steroids, proceeded to surgical resection	11 months, no recurrence
Tublin 2007 [6]*	7 cases include 5 females and 2 males, ages 29-68. All presented with obstructive jaundice. 3 presented with RUQ pain.	-	Hilar	ERCP brushings in all cases - inconclusive	-	Surgical resection in 6 cases. Biliary stents in 1 case	5 cases 1-6 years follow up, no recurrence. 2 cases progressive liver dysfunction biopsied 1 year after surgery, and death occurred in both of these cases (1 case as complication liver biopsy and 1 case acute myocardial infarction)
Kurihara 2007 [28]	69M obstructive jaundice	-	CBD	Per-oral cholangioscopy biopsy - IPT	N/A	Oral prednisolone	3 years, no recurrence
Ashcroft 2009* [29]	59F epigastric pain, obstructive jaundice, and weight loss	-	CHD + GB + pancreas	ERCP brushing/ biopsy – negative malignancy	Cholangiocarcinoma	Surgical resection	7.5 years, no recurrence
Ashcroft 2009* [29]	61M RUQ pain and obstructive jaundice	-	Hilar + CHD + CD	-	Cholangiocarcinoma	Surgical resection	6 years, no recurrence
Abu-Wasel 2012 [3]	55M obstructive jaundice	High / High	CBD	ERCP brushing – negative malignancy	Cholangiocarcinoma	Surgical resection	14 months, no recurrence
Subhash 2012 [30]	21F obstructive jaundice, anorexia, and weight loss	High / High	Hilar	-	Cholangiocarcinoma	Surgical resection	-
Koizumi 2013 [31]	62F asymptomatic mass	Normal/Normal	CBD	Transpapillary biopsy – IPT	-	Oral prednisolone	4 months, no recurrence
Vasiliadis 2014 [32]	71F anorexia, weight loss, and obstructive jaundice	High/High	CBD	Unsuccessful attempt at biopsy	Cholangiocarcinoma	Surgical resection	8 months, no recurrence
Rastogi 2015 [33]	62M obstructive jaundice	High/High	Hilar	-	Cholangiocarcinoma	Surgical resection	6 months, no recurrence
Koiwai 2021 [34]	71M obstructive jaundice	High/High	Hilar	EUS-FNB - IPT	N/A	Oral steroids	-
Current study	52F deranged LFT	Normal/High	Hilar + CHD + CBD + CD	-	Cholangiocarcinoma	Surgical resection	4 months, no recurrence

Clinical Presentation 

The most common presentation was obstructive jaundice, present in 79% (n=26/33) of cases followed by abdominal pain present in 39% (n=13/33) cases, most often localized to the right upper quadrant (n=7). Less commonly (in order of frequency), patients presented with weight loss, anorexia, fever, nausea, lethargy, vomiting, and diarrhea. Where reported, bilirubin was elevated in 77% (n=17/22) of cases and liver function tests were deranged in 91% (n=21/23) of cases.

The liver hilum was the most common location for pseudotumors (n=24); however, it was also reported at the common bile duct (n=12), common hepatic duct (n=5), and cystic duct (n=3). Three cases reported serum immunoglobin G subclass 4 (IgG4) to be elevated; two were diagnosed with autoimmune pancreatitis and concurrent IPT, and one was diagnosed as IgG4-related IPT without systemic disease but with suspected obliterative phlebitis. Only one case described the involvement of concurrent organ systems; Ikeda et al. (1990) described a case involving the lung and bilateral cervical and inguinal lymph nodes [19]. One patient died at presentation prior to intervention due to presumed sepsis, with fungi (unspecified culture) isolated from the pseudotumor at autopsy. No cases identified bacterial or acute viral infections as potential causes of IPT.

Preoperative Workup 

Where reported, most cases had an abdominal computerized tomography scan (81%, n=25/31) or abdominal ultrasound (65%, n=20/31) as the initial imaging modality, with 45% of cases undergoing both (n=14/31). MRI was used in 26% (n=8/31) cases. ERCP was performed in 20 cases, however, endoscopic ultrasound was reported less frequently (n=6). Tumor markers, including CEA, CA 19-9, and/or AFP, were reported in nine cases and were normal in all but one, which reported a borderline CA 19-9.

Nine cases reported attempted biopsy, of which five confirmed IPT (methods of which included core biopsies, per-oral cholangioscopic biopsy, trans-papillary biopsy, and endoscopic ultrasound with fine-needle aspiration). Of these, three cases were managed with steroids, one with biliary stenting, and one with surgical resection. Of the remaining four biopsies, one was unsuccessful, two were inconclusive (ERCP and core biopsy) and one was negative for malignant cells (ERCP). In 14 cases, a preoperative diagnosis of cholangiocarcinoma was made.

Management

Surgical resection was undertaken in 82% (n=27/33) of cases, including one liver transplantation due to the extent of the disease. In two cases, the indication was for progression of IPT despite a trial of oral steroids. Six cases reported utilization of frozen section intraoperatively, all confirming benign pathology. Of those cases that reported follow-up data, all but one (n=21/22) had no disease recurrence post-resection over a median follow-up period of one year (range 4 months-7.5 years). One death unrelated to IPT was reported.

Two cases were managed definitively with biliary stenting; however, only one commented on follow-up, reporting no disease progression at five years. Additionally, there was one case in which biliary stenting failed, requiring surgical resection. Three cases were successfully treated with oral steroids alone, and two of these studies reported no recurrence at follow-up of four months and three years.

Histology 

The current case we are reporting on is the only case in this series to report patches of xanthogranulomatous inflammation.

Discussion

Hilar and extrahepatic bile duct IPT is a rare benign tumor with only 33 cases reported to the best of our knowledge. While intra-hepatic IPT is becoming increasingly well-described with several review articles [8,35-37], extrahepatic and hilar IPT reviews are fewer and involve smaller cohorts. The largest narrative review by Kaneko et al. included 14 cases [5]. A complicating factor is the evolving distinction between IPT and IMT over the past decade. Previously used interchangeably, IPT and IMT are now recognized as distinct entities [9-11]. This article represents the first to systematically review hilar and extrahepatic bile duct IPT, differentiating it from IMT.

Hilar and extrahepatic bile duct IPT had a median presentation age of 57, in keeping with findings that IPT generally presents in older adults. However, as in other parts of the body, it can still occur in childhood, with the youngest case reported being six years old [10,38]. Most patients present with symptoms and signs of obstructive jaundice with concurrent elevation of bilirubin and liver enzymes on blood tests. Abdominal pain, weight loss anorexia, and lethargy are less common symptoms. The liver hilum is the most frequent site of involvement, followed by the CBD.

Most cases were initially investigated with ultrasound (US), CT, or both, with nearly half also undergoing MRI. Where recorded, the preoperative diagnosis was often malignancy, with cholangiocarcinoma being the most common presumptive diagnosis. This aligns with findings that IPT is indistinguishable from malignant lesions on imaging [6].

The pathogenesis of IPT remains unclear; however, several mechanisms have been proposed [12]. Infectious agents, including bacteria and viruses, have been implicated in IPT of other organs [7,39]. Portal venous infection and obliterating phlebitis may lead to recurrent cholangitis, which may contribute to the development of IPT [40-41]. Only one case identified an infectious agent (unspecified fungi) in hilar or extrahepatic IPT [21]; however, a few cases reported associated cholangitis or obliterative phlebitis [17,23].

IgG4-related IPTs are a recognized subtype, characterized by dense infiltration of IgG4-positive plasma cells. These can be associated with IgG4 sclerosing systemic diseases, as in two cases included in this review where the patients had concurrent autoimmune pancreatitis, however, can occur sporadically as was seen in one case [42-43].

Preoperative biopsy confirmed IPT in five out of nine cases. Of these, three cases were subsequently successfully managed with systemic steroids, one with biliary stenting, and one required surgical resection after failing steroid management. In all patients who were successfully managed with steroids, all reported elevated serum IgG4, whereas it was not reported in the case that failed steroid management. Kaneko et al. reported a case where IPT was confirmed with biopsy during exploratory laparotomy, which ultimately proceeded to surgical resection over two years later after failed conservative management with stenting and steroids due to disease progression [5]. IgG4 was also not reported in this case. As such, in some cases, preoperative biopsy may be helpful to avoid surgery, especially in cases of reported elevated serum IgG4.

However, this must be weighed against the risks of biopsy, including potential seeding of malignant pathology, which is difficult to quantify but clinically significant [44-45]. Additional risks include inadequate tissue sampling, leading to false-negative results. In non-cirrhotic patients, hepatectomy is well-established as safe and avoids these risks [46]. Therefore, proceeding to surgery without biopsy is often considered the treatment of choice.

Gohy et al. presented two cases where IPT was diagnosed without tissue biopsy based on clinical findings alone; specifically non-progression of disease, spontaneous disease resolution, and/or response to systemic steroids. Interestingly serum IgG4 was not included in their workup. This approach comes with the risk of misdiagnosis of malignancy, potentially delaying appropriate treatment [46].

Most cases underwent surgical resection, with IPT confirmed on histology. In all cases, a frozen section was used; it was negative for any malignancy. In one case, it led to a more limited resection [32]. Long-term outcomes remain unclear, with a median follow-up of one year. Further research is needed to determine the most effective treatment. In other organ systems, complete IPT resection is associated with good long-term prognosis and low recurrence rates [47].

Histologically, IPT is characterized by a non-specific inflammatory infiltrate consisting of lymphocytes, plasma cells, spindle cells, eosinophils, and macrophages. Someren proposed three histological subtypes: xanthogranuloma-type, plasma cell granuloma-type, and sclerosing-type lesions [40]. The present case is unusual, given it is the only IPT involving porta hepatis and/or extra-hepatic bile duct to report xanthogranuloma-type lesions.

A limitation of this review is the exclusion of studies that reported IMT, which may have included true IPT cases mislabeled due to previous nomenclature. However, given IPT's distinct clinical presentation and disease course, this approach was preferred to avoid including IMT cases that could skew findings on presentation and management.

## Conclusions

IPT at the porta hepatis and extrahepatic bile ducts is rare and typically presents with obstructive jaundice. It is difficult to distinguish it from malignant lesions on clinical presentation and imaging, and so diagnosis typically relies on tissue sampling. Surgical resection is the mainstay of treatment, however, in select cases, especially where serum IgG4 is elevated, a preoperative biopsy may be able to confirm the diagnosis and avoid surgery. However, this must be weighed against the risk of biopsy, including potential tissue seeding, if the underlying lesion is malignant.
